# CircAMOTL1 RNA and AMOTL1 Protein: Complex Functions of *AMOTL1* Gene Products

**DOI:** 10.3390/ijms24032103

**Published:** 2023-01-20

**Authors:** Joanna Sadlak, Ila Joshi, Tomasz J. Prószyński, Anthony Kischel

**Affiliations:** 1 Laboratory of Synaptogenesis, Łukasiewicz Research Network—PORT Polish Center for Technology Development, Stabłowicka 147, 54-066 Wrocław, Poland; 2 Department of Molecular Physiology and Neurobiology, University of Wrocław, Sienkiewicza 21, 50-335 Wrocław, Poland

**Keywords:** AMOTL1, circAMOTL1, circular RNA, backsplicing, cancer, wound healing, heart repair

## Abstract

The complexity of the cellular proteome facilitates the control of a wide range of cellular processes. Non-coding RNAs, including microRNAs and long non-coding RNAs, greatly contribute to the repertoire of tools used by cells to orchestrate various functions. Circular RNAs (circRNAs) constitute a specific class of non-coding RNAs that have recently emerged as a widely generated class of molecules produced from many eukaryotic genes that play essential roles in regulating cellular processes in health and disease. This review summarizes current knowledge about circRNAs and focuses on the functions of AMOTL1 circRNAs and AMOTL1 protein. Both products from the AMOTL1 gene have well-known functions in physiology, cancer, and other disorders. Using AMOTL1 as an example, we illustrate how focusing on both circRNAs and proteins produced from the same gene contributes to a better understanding of gene functions.

## 1. Introduction

Circular RNAs (circRNAs) are single-strand RNAs containing exons, introns, or both, which have a form of covalently closed circles. They are produced during alternative splicing, i.e., backsplicing, of the pre-mRNAs of many eukaryotic genes. Historically, circRNAs were identified for the first time by Sanger and colleagues in 1976 [1]. Despite the identification of circRNAs, they were poorly studied for decades, mainly because they were hypothesized to be byproducts of alternative splicing defects with poor or absent functional potential [2,3]. It was only during the last decade that circRNAs became a research hotspot and the number of publications began to increase exponentially. The focus on this new research area emerged from several pioneering papers showing that thousands of circRNAs are ubiquitously expressed [4,5,6,7,8]. Indeed, with the emergence of high-throughput sequencing, Jeck and colleagues found that more than 25,000 distinct circRNA species are potentially present in human fibroblasts [5]. These circRNAs represent 14.4% of actively transcribed genes in the human genome. Moreover, circRNAs are highly conserved as they have been shown to be expressed across eukaryotes [9]. Due to their high stability and resistance to exonucleases, many circRNAs are found in higher amounts in cells than linear mRNAs produced from the same transcripts [4]. The majority of the circRNAs are composed of exons; however, it has been suggested that 19% of circRNAs contain only intronic sequences, and 5% are from intergenic regions [10]. At the functional level, circRNAs have been shown to regulate gene expression, signal transduction, protein, and RNA stability. They regulate many important processes such as development, stem cell differentiation, cell proliferation and migration, wound repair, cancer development, and metastasis (reviewed by Lin and colleagues [11]). The most commonly described mechanism of action of circRNAs involves sequestering microRNAs in the cytoplasm through base pairing [7]. CircRNAs were also found to bind many proteins and could serve as a scaffold facilitating protein–protein interactions or promoting their nuclear translocation [12,13]. Finally, the misregulation of circRNAs has been associated with human diseases such as cancers, cardiovascular diseases, and neurodegenerative disorders, and they have been proposed as potential promising biomarkers [14,15,16,17].

CircRNAs produced from the Angiomotin-like 1 (AMOTL1) gene provide an example of a variety of functions that this class of molecules could serve in health and disease, particularly in cancer. Similarly, AMOTL1 protein encoded by the same transcript is implicated in various cellular processes, including controlling signaling pathways (e.g., Wnt/β-catenin and Hippo/YAP), regulating actin cytoskeleton, gene expression, cellular junctions, mechanotransduction, and angiogenesis [18,19]. Our review describes the complexity of AMOTL1 gene functions and points to the importance of analyzing both proteins and circRNAs that can be produced from the same locus.

## 2. AMOTL1—From Gene to Protein

### 2.1. AMOTL1 and Angiomotin Proteins

**Figure 1:**
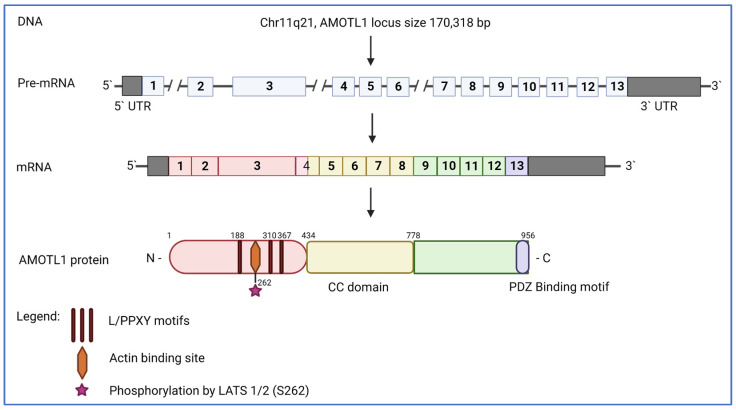
Schematic representation of AMOTL1 gene, mRNA, and protein. *AMOTL1* locus, located on chromosome 11q21 of the human genome, comprises 13 exons. AMOTL1 protein contains L/PPXY motifs, an actin-binding site, and a phosphorylation site modified by LATS1/2 within the ID domain (orange). The coiled-coil domain is located in the middle part (yellow) and is followed by a C-terminal fragment (green) containing the PDZ-binding motif (purple).

Angiomotin-like1 protein (or AMOTL1) belongs to the Angiomotin family of proteins (also called Motins) produced from three genes, Angiomotin (*AMOT*), Angiomotin-like1 (*AMOTL1*), and Angiomotin-like2 (*AMOTL2*). AMOTL1 was originally discovered as a junction-enriched and associated protein (JEAP) expressed in tight junctions [20,21]. Like other family members, this protein has the intrinsically disordered (ID) domain located in the N-terminal part, which contains actin-binding motifs and sequences that interact with the Yes1-Associated Transcriptional Regulator (YAP) (Figure 1). The ID sequence is phosphorylated by LATS1/2, a kinase involved in the Hippo signaling pathway regulating YAP activity, and this modification likely blocks interactions with the actin cytoskeleton and affects certain AMOTL1 functions [22]. Next to the ID domain is a coiled-coil region responsible for homo- and hetero-oligomerization of Motins as well as interactions with various other binding partners [20,23]. The C-terminal part of AMOTL1 contains a PDZ-binding motif, which is responsible for interactions with several PDZ domain-containing proteins [24].

Angiomotins are widely expressed by many cell types and in different tissues. The AMOTL1 protein plays important roles in regulating cell division, cell junctions, and other cellular functions in physiological and pathological conditions, especially in cancer. Therefore, AMOTL1 and other Motins are important targets from a therapeutic perspective.

AMOTL1, like other full-length isoforms of Angiomotins, binds to F-actin through the F-actin binding region in the N-terminal domain. AMOTL1 localization in many cells correlates with phalloidin staining, and it partially colocalizes with accessory proteins of stress fibers such as Myosin II, which indicates a role for AMOTL1 in organizing the cellular architecture. It is, therefore, not surprising that AMOTL1 knockdown leads to deficits in cell migration speed and a lack of directional movement [23]. In epithelial cells, AMOTL1 is located in the tight junctions, where it interacts with junctional proteins, e.g., MAG-1, ZO-1, PATJ, SYX, and MUPP1 [25,26,27]. Post-translational modification of AMOTL1 is important to regulate AMOTL1 activity at the cell–cell junction. AMOTL1 mono-ubiquitination by HECW2 ligase is required to maintain AMOTL1 at junctions and protects junctional integrity [28]. AMOTL1 can also be polyubiquitinated by NEDD4L, leading to its degradation. At the same time, interactions with YAP, which recruits c-Abl tyrosine kinase, could inhibit AMOTL1 degradation through the phosphorylation of NEDD4L, leading to the stabilization of tight junctions [29].

All Angiomotins play important roles in angiogenesis. In vitro studies have shown that the family members regulate the paracellular permeability of endothelial cells through the formation of heterodimer complexes composed of AMOT and AMOTL1 [20]. In addition, a study in zebrafish revealed that Amotl1 facilitates endothelial tip cell migration, promoting vessel formation [20]. Another study showed that AMOTL1 acts as a scaffold to connect N-cadherin of endothelial cells and pericyte cells to generate mechanical force via actin filaments and therefore maintain the junction complex required for vascularization of the mouse retina [30].

AMOTL1, like the other members of the Motin family, has been shown to inhibit the Wnt/β-catenin pathway [31]. Knockdown of *amotl2* in zebrafish leads to embryonic dorsalization, a phenotype associated with the Wnt/β-catenin pathway. Moreover, Angiomotin family members are able to inhibit WNT/β-catenin activity also in mammalian cells in vitro. Mechanistically, Amotl2 has been shown to bind with β-catenin and sequester it to the Rab11-positive recycling endosomes, reducing the amounts of free β-catenin in the cytosol and in the nucleus [31]. Similar functions have also been proposed for AMOTL1.

Another broad function of all Angiomotins is to regulate the Hippo signaling pathway. The core of this pathway consists of two kinases, MST1/2 and LATS1/2, which regulate the phosphorylation of the transcription co-activator YAP. Phosphorylation of YAP prevents its migration to the nucleus and blocks its transcriptional activity and expression of many Hippo pathway-controlled genes. Angiomotin binding to YAP sequesters it in the cytoplasm, preventing both its nuclear migration and expression of YAP-dependent genes. There are also examples of the opposite mechanisms, in which Angiomotin binding to YAP promotes its nuclear migration and transcriptional activity. For instance, in myocardial cells, the cadherin junction protein FAT4 sequesters AMOTL1 at cell contacts and prevents its interactions with YAP. This inhibits YAP translocation to the nucleus, suppresses gene expression, and restricts cell growth in the myocardium [32]. Components of the Hippo pathway are also known to regulate Angiomotins, and Motins can influence Hippo pathway activity at many levels. For instance, the LATS1/2 have been shown to phosphorylate Angiomotins, which can regulate their association with actin cytoskeleton and YAP activity [22,33,34,35]. Angiomotin binding has also been shown to stimulate LATS1/2 autophosphorylation and can serve as a scaffold on which LATS1/2 interact with downstream YAP and upstream MST1/2 [36]. Moreover, LATS1/2 proteins can be activated by another kinase called Merlin, and Angiomotin binding to Merlin releases it from its autoinhibitory state and promotes its association with LATS1/2 kinases [37]. Merlin activity, on the other hand, can be regulated by mono-ubiquitination by NEDD4L, and Amotl1 serves as a scaffold, recruiting the ubiquitin ligase to its target [38].

The complex functions of Angiomotins additionally illustrate their role in cancer cells, where they can act either as tumor suppressors or activators. For instance, AMOTL1 protein expression increases in glioma and gastric cancer, where it protects YAP from degradation, which stimulates the expression of proto-oncogenic genes such as connective tissue growth factor (CTGF) [39,40]. In glioma, the inhibition of AMOTL1, both in vitro and in vivo, has an anti-tumor effect. This study also indicates that AMOTL1 could serve as a diagnostic marker and a potential target for glioma treatment. Moreover, AMOTL1 protein expression also correlates with the progression of breast cancer aggressiveness and metastasis by stimulating Src activity [41]. The report also shows that the loss of Merlin could be one of the reasons for the upregulation of AMOTL1 as Merlin negatively regulates AMOTL1 activity by mediating its phosphorylation, therefore facilitating the interaction with the Nedd4 family leading to its proteasomal degradation. On the other hand, AMOT and AMOTL2 have also been observed to have a tumor suppressor role in ovarian cancer and glioblastoma, respectively, where their downregulation in cells avails YAP for its nuclear function and enhances cell proliferation [42,43].

### 2.2. AMOTL1 Splicing

Alternative splicing is a powerful mechanism through which cells regulate the expression and function of many transcripts. *AMOTL1*, like other Angiomotins, are reported to undergo alternative splicing and give rise to at least two isoforms, where the deletion of either exon 2 or both exons 2 and 3 have been observed [44]. It has been reported that the deletion of exon 2 does not change the reading frame and the protein localizes similarly to full-length AMOTL1. However, the deletion of exons 2 and 3 gives rise to a premature stop codon. The production of such a truncated protein has not been observed, and this spliced mRNA is restricted to certain tissues. Furthermore, another study showed a different isoform of AMOTL1 that lacks 86 amino acids from the N-terminal region [20]. Recently, it became apparent that *AMOTL1* pre-mRNA can be alternatively spliced to generate circular RNA, which has important functions in several cell types, as discussed in Section 4.

## 3. Circular RNAs

### 3.1. Mechanisms of circRNA Biogenesis

Many eukaryotic genes undergo alternative splicing, which removes certain exons from pre-mRNA. Alternative splicing for a given gene can occur in all cells, or only in particular cell types or tissues and in specific physiological or pathological states. Alternative splicing can produce functionally different protein isoforms. It is estimated that alternative splicing occurs in 95% of human transcripts with multiple exons [45]; therefore, pre-mRNA processing contributes significantly to the diversity of the proteome.

**Figure 2:**
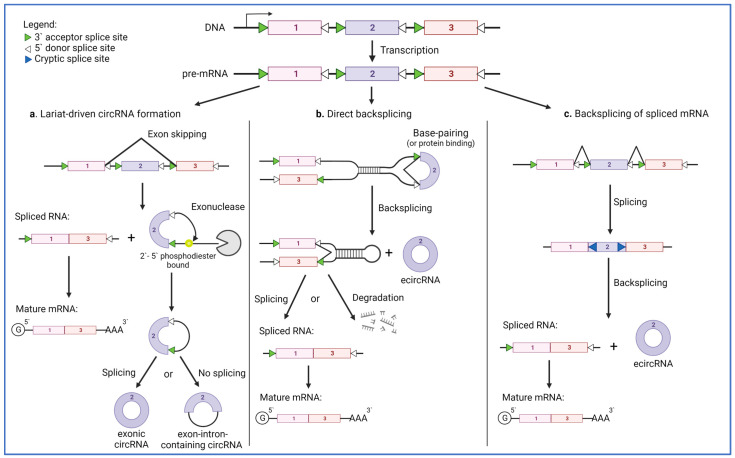
Mechanisms of circRNA biogenesis. CircRNAs can be generated from linear RNAs by three different mechanisms. (**a**) In the lariat-driven process, the pre-mRNA is alternatively spliced by skipping exon 2. This process gives rise to a spliced transcript containing exons 1 and 3 and a circular form of RNA called a lariat. The lariat is generated by RNA branching through a 2′-5′ phosphodiester bound. The free RNA end is next digested by an exonuclease. The newly formed circRNA can undergo additional splicing, leading to the excision of the intron and producing an exonic circRNA (ecircRNA), or it could remain as an exon-intron-containing circRNA (EIciRNA). (**b**) In the second mechanism, backsplicing occurs before conventional splicing. In this case, base pairing or the association with RNA-binding proteins brings into close proximity the exon 2-flanking splice sites. The first splicing event forms the circRNA containing exon 2, and the rest of the pre-mRNA can undergo additional splicing or be degraded. (**c**) The third mechanism has been described only in cancer and involves the backsplicing of mature mRNA, which utilizes cryptic splice sites within the mRNA.

Recently, it has been demonstrated that a large number of transcripts can undergo so-called reverse splicing (or backsplicing). This process involves ligation of the acceptor splice site to the donor located downstream (instead of upstream) and leads to RNA circularization (Figure 2). Backsplicing is facilitated when certain splicing sites are in close proximity, and this can be regulated either by base pairing between sequences within the RNA molecule or by RNA-binding proteins. Two major mechanisms of circRNA formation, namely direct-backsplicing and lariat-driven backsplicing, have been recognized; these differ in the order of splicing events. If conventional splicing occurs first, it could lead to the removal of linear RNA containing exons, introns, and splice sites [46]. The interaction of sequences within the released fragment may lead to the formation of a so-called lariat, which brings two splicing sites into close proximity, promoting the backsplicing event (Figure 2a) [47]. In the alternative scenario, backsplicing occurs before conventional splicing. Such an event could be facilitated by RNA sequence pairing or RNA-binding proteins and is called direct backsplicing (Figure 2b) [48,49]. The important difference is that, during direct backsplicing, the rest of the pre-mRNA can undergo conventional splicing to produce functional mRNA and protein or is degraded. Therefore, the mechanism for circRNA formation can be linked to the downregulation of mRNA and protein synthesis. Currently, it is believed that splicing and backsplicing are coupled events or even competing processes, and various factors can affect the outcome. There is also a third mechanism for circRNA formation, reported so far only in cancer, involving re-splicing of a mature mRNA [50]. In this mechanism, the initial splicing removes the canonical splice sites, and the backsplicing event and circularization of RNA occur at cryptic splice sites in the mature mRNA (Figure 2c).

### 3.2. Regulation of circRNA Expression

There are many factors that could influence circRNA biogenesis, reviewed in detail by Li and colleagues [51]. Generally speaking, circRNAs are generated in a process of alternative splicing of pre-mRNA. Therefore, gene expression and the rate of pre-mRNA formation play important roles in the regulation of circRNA production. Interestingly, it has been proposed that the transcription elongation rate and pausing of polymerase II could be important events that affect pre-mRNA processing, including splicing [52]. Transcriptional pausing could influence the folding of partially synthesized RNA and, therefore, alter the internal pairing that promotes circRNA generation. On the other hand, some studies have suggested that most backsplicing occurs after transcription has been completed [53]. Backsplicing depends on factors that work in cis and in trans to the pre-mRNA. The sequences that promote RNA pairing are encoded within the transcript, often located in long introns flanking the exon.

It has been shown that complementary Alu repeats or complementary inverted repeat sequences located in intronic loci promote the formation of a transient stem-loop structure, allowing the positioning of a distal donor splice site close to a proximal acceptor splice site [54]. Furthermore, intronic complementary sequences as short as 30–40 nucleotides have been shown to be sufficient to allow base pairing, followed by circularization of the RNA [55]. Long repeats preferentially lead to co-transcriptional production of circRNA, and short repeats are mostly in favor of post-transcriptional biogenesis of circRNA. Interestingly, using *Drosophila* minigenes, it has been shown that circRNA biogenesis and pre-mRNA canonical splicing compete against each other, and the outcome, i.e., the production of circRNA versus a linear mRNA counterpart, is tissue-specific [56]. The splicing machinery and RNA-binding proteins (RBP) are the trans elements that facilitate circRNA production.

Most of the RBPs involved in the production of circRNAs are proteins that regulate RNA splicing (reviewed in [57]). Among them, Quaking is a critical regulator of circRNAs production during human epithelial-mesenchymal transition (EMT) [58]. Hundreds of circRNAs have to be dynamically regulated to allow EMT, and Quaking governs the production of over one-third of these circRNAs. Other splicing factors control the generation of specific circRNAs. For instance, NOVA1 mediates the production of circUVRAG in vascular smooth muscle cells, regulating cellular adhesion and migration [59]. Another example described later in this review is RNA binding motif 25 (RBM25) regulating circAMOTL1 in prostate cancer [60].

Epigenetic modifications regulate many aspects of RNA metabolism, processing, and function. Over 160 types of co-transcriptional changes have been identified in RNAs. Among them, the N^6^-adenosine methylation (m6A) is the most abundant and has been extensively studied (reviewed in [61,62]). Functionally, m6A regulates RNA decay and stability, miRNA biogenesis, RNA translocation and subcellular localization, gene expression, and pre-mRNA splicing. Recently, similar modifications have been discovered on circRNAs (reviewed in [63]). Genome-wide mapping in human embryonic stem cells (hESC) and HeLa cells identified that m6A modifications of circRNAs are widespread. Interestingly, different residues were modified within the shared sequences in mRNA and circRNAs produced from the same transcripts. Additionally, some of these modifications were cell-type-specific [64]. Similarly, different cell types express various sets of RBPs and proteins modifying the m6A epigenetic marks on RNA. Collectively, heterogeneity in RBPs expression and m6A modifiers in the cells provides an interesting mechanism for how circRNAs can be regulated in a cell-type-specific manner.

There are also known mechanisms that inhibit circRNA production. For instance, adenosine deaminase acting on RNA (ADAR) recognizes double-stranded RNA and mediates A-to-I editing on Alu repeats, thereby disrupting base pairing and inhibiting circRNA biogenesis. Finally, it has been shown that the rate of backsplicing and circRNA formation depends on the availability and the expression of the spliceosome machinery. Liang et al. have reported that the knockdown or pharmacological inhibition of spliceosome components promotes circRNA formation instead of linearly spliced RNA products [65]. A similar effect on circRNA biogenesis was found with the inhibition of RNA polymerase II termination [65]. Altogether, these results demonstrate that circRNA production, compared to its linear counterpart, is favored when the pre-mRNA processing machinery is limited. To summarize, the biogenesis of circRNAs is a complex process that can involve various reactions; a large number of trans and cis-acting factors can influence it. Collectively, these processes, similarly to canonical alternative splicing, contribute to the generation of a vast number of different RNAs in a cell type- and tissue-specific manner, which additionally can be regulated differently in development, adulthood, aging, and disease. The last level of circRNA regulation depends on the rate of their degradation (reviewed by Guo et al. [66]). As circRNAs constitute a covalently closed circular form of RNA, which does not have free ends, these structures are resistant to exonucleases. Four mechanisms are described that facilitate circRNA degradation. The first step involves linearization via endonuclease attack, which is then followed by degradation via the exosome complex or 5′-3′exoribonuclease. Several proteins recruited to circRNAs or RNA sequence modification marks have been identified to allow circRNA targeting by endonucleases. For instance, Hansen and colleagues proposed miRNA-dependent circRNA degradation by Argonaute 2 (Ago2) cleavage [67]. Moreover, different marks can be recognized in favor of circRNA degradation, such as N^6^-adenosine methylation [64,68] or RNA secondary structures, such as double-strained RNA or highly-structured RNAs [69,70]. Finally, another mechanism is hypothesized to regulate intracellular levels of circRNAs through extracellular vesicles. Indeed, circRNAs were found to be carried in these vesicles together with other cellular components such as proteins, lipids, and RNAs [71]. Secretion of circRNAs in exosomes is a possible mechanism for circRNA clearance; however, it is also a way to transfer functional circRNAs for cell-to-cell communication. Furthermore, circRNAs have been proposed to be used as biomarkers by analyzing their levels present in fluids such as blood and urine in several diseases, such as in cancer [72], in neurodegenerative and neuropsychiatric disorders (reviewed in [14,73]), and in cardiovascular disease [74].

### 3.3. CircRNA Functions

The main function of circRNAs is to regulate gene expression at the transcriptional and post-transcriptional levels [6], and it could involve interactions with other non-coding RNAs or proteins. At the post-transcriptional level, circRNAs bind to specific sets of microRNAs, thereby preventing their association with target mRNAs and protecting linear mRNA from degradation or translational inhibition [7,75]. This mechanism of action is called microRNA sponging. Circular RNAs are also known for their ability to interact with many proteins, changing their functions, intracellular localization, and stability. For instance, binding to certain transcription factors or signaling molecules could trigger their translocation to the nucleus and efficient depletion from the cytoplasm. If this involves transcription machinery (e.g., c-MYC or STAT3), this could directly affect gene expression. It has also been shown that certain circRNAs have the ability to bind to RNA polymerase II, thereby modulating its transcriptional activity [76]. Nuclear targeting of signaling molecules changes the repertoire of available proteins for interaction and affects post-translational modifications, which, as in the case of phosphorylation, often regulate protein function. Finally, targeting cytoplasmic proteins into the nucleus could protect them from the degradation machinery available in the cytosol. CircRNAs can also serve as a scaffold for proteins, promoting their interactions if two or more of them bind to a circRNA simultaneously. Interestingly, there are also reports showing that circRNAs can be translated into so-called circ-proteins in a 5′ cap-independent manner using either an internal ribosomal entry site or m6A modifications to initiate its translation (reviewed in [77,78]). To date, however, only a handful of polypeptides produced from circRNAs have been identified, and little is known about the abundance of such circRNA-derived proteins or their functions. Most of the peptides translated from circRNAs have been shown in cancers (reviewed in [79]), but translation of circ-ZNF609 has been reported in myoblasts, with a role in regulating their proliferation [80].

It is clear that the expression of many genes leads to the production of a large number of circRNAs that play important cellular functions. In the next sections, we will summarize the current knowledge on AMOTL1 circRNAs that play important roles in many physiological and pathological processes and greatly contribute to the functions of other *AMOTL1* gene products (both mRNA and protein). These findings are summarized in Table 1.

## 4. The Functions of circAMOTL1

**Figure 3:**
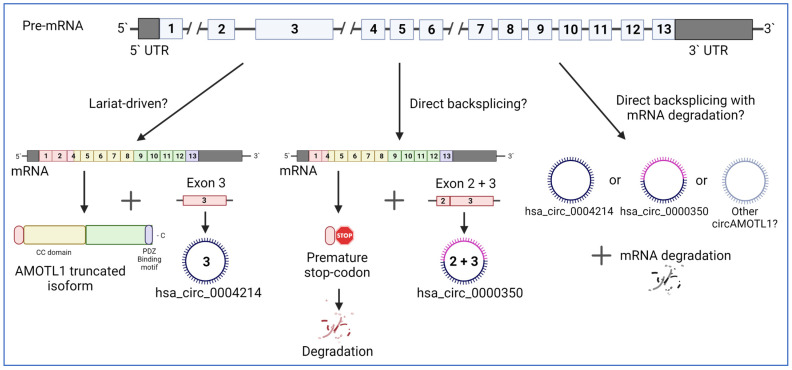
Hypothetical models of AMOTL1 circRNAs production. A lariat-driven mechanism could produce circAmotl1 containing only exon 3 (hsa_circ_0004214) during RNA splicing, leading to the production of the truncated protein. On the other hand, the direct backsplicing could lead to the production of circAMOTL1 with exons 2 and 3 (hsa_circ_0000350). The other product from this process is mRNA with a premature stop codon, which does not allow for AMOTL1 protein synthesis. The alternative hypothesis would include direct backsplicing with the degradation of the remaining mRNA.

To date, two main circAMOTL1 RNAs are described in the literature that differ in their exon composition. These are hsa_circ_0004214, which contains only exon 3, and hsa_circ_0000350, composed of exons 2 and 3. Their length is 922 and 1072 bases, respectively (USCS genome browser or CircInteractome). However, at least three additional uncharacterized circAMOTL1 were identified in human—hsa_circ_0096833, hsa_circ_0024049, and hsa_circ_0024050 [4,8]. The exact mechanism allowing the production of these circAMOTL1 is unknown but hypothetical mechanisms are depicted (Figure 3). In this section, we will summarize known functions and mechanisms of action for circAMOTL1.

### 4.1. CircAMOTL1 Mediates c-myc Nuclear Translocation to Promote Breast Cancer Progression

**Figure 4:**
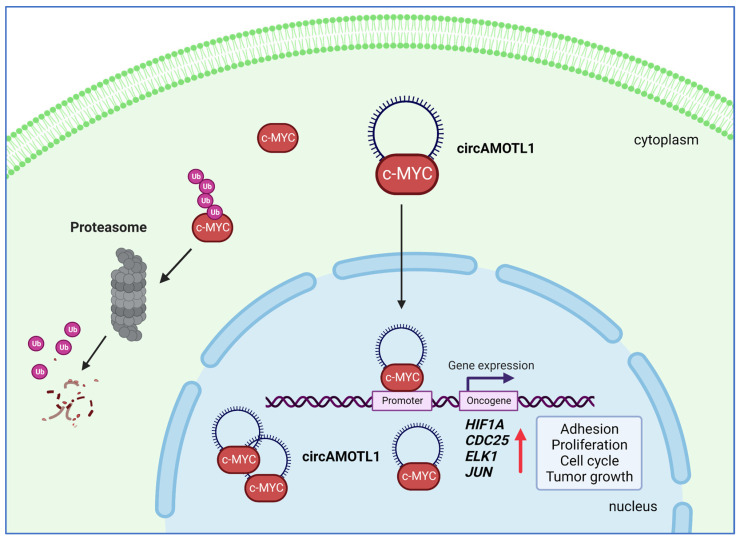
In breast cancer cells, circAMOTL1 binds c-MYC protein in the cytoplasm, facilitating translocation of the complex to the nucleus. In the nucleus, circAMOTL1 enhances the binding affinity of c-MYC to multiple oncogene promoters (e.g., *HIF-1α*, *CDC25*, *ELK1*, *JUN*), promoting their expression and cancer growth. At the same time, interactions between circAMOTL1 and c-MYC prevent c-MYC degradation in the cytoplasm and enhance c-MYC stability in the nucleus.

Breast cancer is the most commonly diagnosed cancer, with more than 2 million cases and almost 690,000 deaths annually worldwide [88]. Yang et al. [81] showed that levels of circAMOTL1 (hsa_circ_0004214; generated from the largest, third exon of *AMOTL1* gene) were significantly elevated in tumor samples isolated from breast carcinoma. Similarly, the levels of circAMOTL1 were high in several breast cancer cell lines (i.e., MB-231, H460, SK-BR-3, HTB-126, MCF-7) [81]. Interestingly, the expression of circAMOTL1 was dependent on cell density, which decreases in over-confluent cells. This suggested that circAMOTL1 is involved in proliferation control. Silencing circAMOTL1 expression indeed decreased proliferation and survival in two breast cancer cell lines—MB-231 and SK-BR-3. Likewise, the ectopic overexpression of circAMOTL1 increased proliferative capacity. Importantly, *AMOTL1* mRNA and protein expression was not influenced by altered levels of circAMOTL1 RNA. To confirm these in vitro observations, Yang et al. [81] subcutaneously injected MDA-MB-231 or HepG2 cells overexpressing circAMOTL1 into immune-compromised mice. Tumor formation and malignancy were enhanced to the point that the invasive cells infiltrated muscles and bones. Interestingly, unlike in several other cell types, in breast cancer cells, circAMOTL1 was found predominantly in the nucleus. Therefore, it is rather unlikely that circAMOTL1 regulates microRNA in breast carcinoma since, for miRNA sponging, circRNAs have to be present in the cytoplasm. In the next set of experiments, the authors demonstrated that circAMOTL1 binds to several oncogenic proteins, i.e., c-MYC, EGF, c-MYB, NF1, and AKT. c-MYC showed the strongest binding to circAMOTL1 and translocated into the nucleus in a circAMOTL1-dependent manner. Nuclear translocation of c-MYC, in turn, enhanced the expression of the c-MYC-dependent genes *HIF-1α*, *Cdc25a*, *ELK-1*, and *JUN*. The oncogenes HIF-1α and ELK-1 promote cancer progression by regulating gene expression [86,89,90], while CDC25a affects the cell cycle [91], and JUN facilitates tumor growth [92]. These results suggest that, in breast cancer cells, circAMOTL1 acts as a scaffold for c-MYC protein, facilitating its nuclear translocation (which also protects c-MYC from degradation), upregulating c-MYC targets, and, as the outcome, promoting cancer growth (Figure 4). Similar malignant behaviors in circAMOTL1-overexpressing MDA-MB-231 cells treated with PAX (a first-line drug for metastatic breast cancer treatment) were reported in another study. Ma et al. [82] suggested that circAMOTL1 is involved in developing chemoresistance against PAX treatment through upregulating phosphorylated AKT levels and its downstream pro-survival genes.

### 4.2. CircAMOTL1 Promotes Cervical Cancer Progression through miRNA Sponging and Protecting AMOTL1 and SIK2 mRNA from Degradation

**Figure 5:**
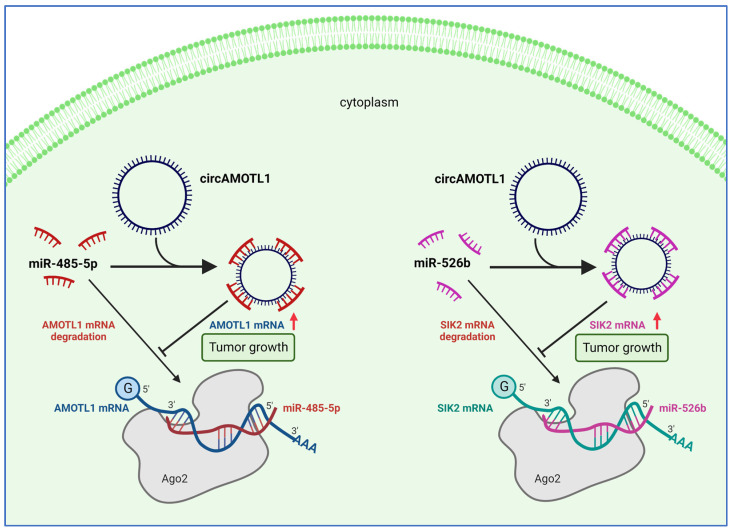
In cervical cancer cells, circAMOTL1 is concentrated in the cytoplasm, where it serves as a sponge that sequesters different miRNAs. miR-485-5p targets *AMOTL1* mRNA, while miR-526b degrades *SIK2* mRNA. CircAMOTL1, therefore, promotes cervical cancer progression by inhibiting miRNA-dependent degradation of oncogene mRNAs such as *AMOTL1* and *SIK2*.

Cervical cancer is the fourth most prevailing cause of mortality in women throughout the world [93]. Like in breast cancer, circAMOTL1 was found to be upregulated in the cervical cancer cell lines C-33A, HeLa, SiHa, and CaSki, and in tumor tissues collected from patients [83,84]. The highest circAMOTL1 levels were observed in metastatic cells that also showed increased expression of *AMOTL1* linear mRNA [83]. Importantly, upregulation of *AMOTL1* mRNA and circAMOTL1 are associated with a poor prognosis in cervical cancer patients. Ou et al. [83] showed that overexpression of circAMOTL1 in cultured CaSki cells increased cell viability, proliferation, and migration. Similarly, subcutaneous injection of circAMOTL1-overexpressing C-33A cells led to elevated tumor sizes and weights, which confirms the oncogenic potential of circAMOTL1 in vivo. It turned out that the enhanced expression of circAMOTL1 and *AMOTL1* mRNA was not coincidental, and it was not simply due to increased *AMOTL1* promoter activity. In CaSki cells, circAMOTL1 was shown to localize predominantly to the cytoplasm, where it functioned as a sponge sequestering miR-485-5p, downregulating *AMOTL1* mRNA levels (Figure 5) [83]. These results are interesting because they show the interplay between the circRNA and its parental gene. Indeed, several other circRNAs (e.g., circMbl), or even proteins translated from circRNAs (i.e., circFGFR1p), were shown to regulate the expression of mRNA or the activity of protein from their host gene, revealing the complexity of the underlying regulatory network [56,94].

Interestingly, in cervical cancer, unlike in breast cancer, the tumor-promoting effect of circAMOTL1 was independent of c-MYC regulation, probably because circRNA was not translocated into the nucleus. On the other hand, in cervical cancer, the function of circAMOTL1 is not limited to the regulation of its own gene expression. Sun et al. [84] reported that cytoplasmic circAMOTL1 works as a sponge for another microRNA, i.e., miR-526b. This miRNA has been shown to inhibit the expression of salt-inducible kinase 2 (SIK2), which is a known oncogene responsible for glucose and lipid metabolism [84,95].

### 4.3. CircAMOTL1 Enhances ENO1 Expression via miRNA Sponging and Promotes Oral Cancer Development

Oral lichen planus (OLP) is a chronic, non-infectious inflammation of the oral mucosa, which can transform into oral cavity squamous cell carcinoma (OCSCC). According to recent reports, it could account for over 170,000 deaths annually worldwide [88,96,97]. Several studies on cancers have indicated that an increased level of ENO1 may be associated with the tumorigenic process [98]. ENO1 is a protein with many cellular functions, as it is a glycolytic enzyme that catalyzes the conversion of 2-phosphoglycerate to phosphoenolpyruvate [99,100]. At the same time, ENO1 has been shown to regulate many signaling pathways involved in cancer development, including PI3K/AKT, AMPK/mTOR, and WNT/β-catenin [101]. ENO1 can also bind to DNA and RNA, with effects on transcription and translation by regulating the *c-MYC* promoter [101]. As in several other cancer types, OCSCC cells collected from patients and the model oral squamous cell carcinoma (OSCC) cell line CAL-27 show increased ENO1 expression. Liu et al. [85] discovered that the increased expression of ENO1 in OSCC specimens corresponds with the upregulation of circAMOTL1, suggesting its involvement in cancer development. Bioinformatics analysis determined that two miRNAs, i.e., miR-22-3p and miR-1294, were the most likely to target both ENO1 and circAMOTL1. Liu et al. [85] proposed a mechanism of OSCC tumorigenesis in which circAMOTL1 enhances ENO1 expression by sponging miR-22-3p/miR-1294, leading to the positive regulation of proteins associated with cell proliferation. However, it needs to be taken into consideration that this study was only a correlation analysis, and a direct functional link between circAMOTL1 and miR-22-3p, miR-1294, and ENO1 awaits further investigation.

### 4.4. CircAMOTL1 Blocks EMT in Prostate Cancer by miRNA Sponging, Increasing the Expression of Protocadherins

**Figure 6:**
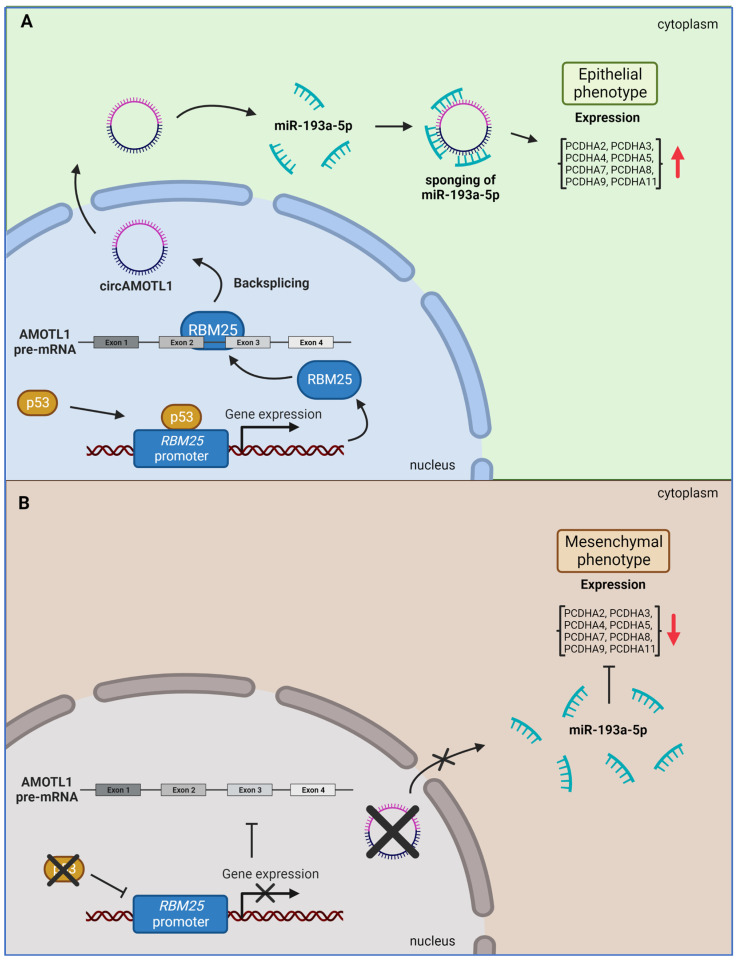
In prostate cancer, metastatic epithelial cells need to undergo an epithelial-to-mesenchymal transition. (**A**) In healthy epithelial cells, the p53 protein promotes the expression of RBM25 RNA-binding protein, which facilitates the formation of circAMOTL1. After translocation to the cytoplasm, circAMOTL1 binds and sequesters miR-193a-5p, acting as a cytoplasmic miRNA sponge. Sponging of miR-193a-5p by circAMOTL1 protects mRNAs of the protocadherin family from miRNA-dependent degradation. This process leads to increased protocadherin production and preserves the epithelial character of enterocytes. (**B**) In primary prostate cancer cells, p53 is either lost or mutated, which inhibits the expression of RBM25. Loss of RBM25 decreases the production of circAMOTL1. In the absence of circAMOTL1, miR-193a-5b targets PCDHA family mRNAs and reduces cellular adhesion. At the same time, increased vimentin and β-catenin expression promotes cell migration and EMT.

CircAMOTL1 has also been shown to promote the development of prostate cancer (PCa), the second leading cause of cancer death among men worldwide, with over 1.4 million cases diagnosed annually [88]. Prostate cancer cells can become metastatic via the process of epithelial to mesenchymal transition (EMT), in which cells lose their ability to adhere to the neighboring cells in the epithelium, lose their attachment to the extracellular matrix (ECM), and start to migrate [102,103]. EMT involves the downregulation of the E-cadherin, a critical player in adherens junctions, and the upregulation of mesenchymal proteins such as β-catenin and vimentin, which facilitate migration [103]. Yang et al. [60] reported that human prostate cancer tissues and the PCa cell lines PC3 and DU145 show significantly decreased expression of circAMOTL1 (hsa_circRNA_000350) that contains exon 2 and exon 3 of the *AMOTL1* gene. These low circAMOTL1 levels appear to be a part of the mechanism regulating tumor cells because overexpression of circAMOTL1 significantly reduced PC3 and DU145 PCa cell line migration and invasion, while knockdown of circAMOTL1 had the opposite effect. The authors proposed that circAMOTL1 regulates prostate cancer metastasis by promoting EMT since increased circAMOTL1 expression led to increased E-cadherin and decreased vimentin and β-catenin levels. Mechanistically, it has been shown that circAMOTL1 works by sponging miR-193a-5p, which critically regulates the expression of protocadherins (PCDHA2-5/7-9/11), a family of cell adhesion molecules. Interestingly, PCDHA family members share a conserved miR-193a-5p-binding site in their 3′ UTR. The authors also investigated the molecular pathway that downregulates circAMOTL1 in PCa. They showed that the p53 protein, which is frequently mutated or downregulated in prostate cancer, promotes the expression of RBM25, an RNA-binding protein that interacts with *AMOTL1* pre-mRNA and promotes circAMOTL1 formation. Thus, in the absence of functional p53, there is decreased production of RBM25, decreased production of circAMOTL1 RNA, and increased free miR-193a-5p, which downregulates the production of protocadherins, thereby promoting EMT (Figure 6A,B).

### 4.5. CircAMOTL1 Promotes Wound Healing by Blocking miR-17-5p Transcription and Increasing DNMT3, STAT3, and Fibronection Expression Levels

**Figure 7:**
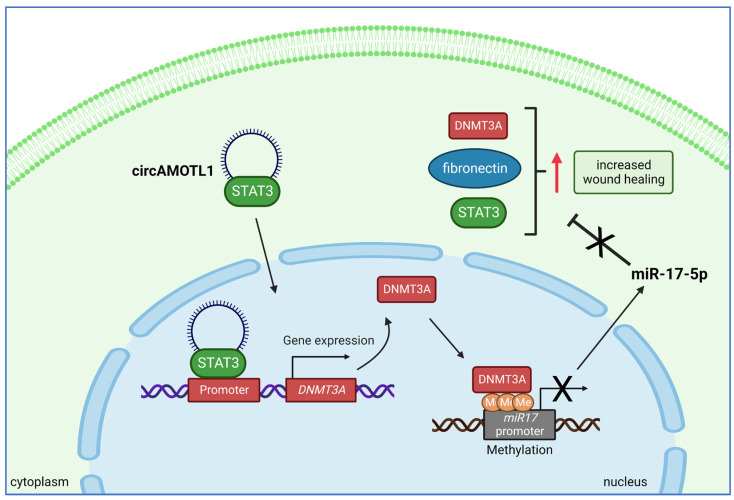
CircAMOTL1 plays an important role in wound healing. During this process, fibroblasts intensively proliferate and migrate into the wounded area. Migrating cells have increased levels of circAMOTL1, which interacts with STAT3 in the cytoplasm and facilitates its translocation into the nucleus. Nuclear STAT3 binds to the regulatory elements of the *DNMT3A* gene, stimulating expression. As a result, DNMT3A binds to the miR-17 promoter and deactivates it by methylation. This leads to decreased levels of miR-17-5p and increased levels of DNMT3A, fibronectin, and STAT3, which promotes wound healing.

As mentioned in the previous sections, circAMOTL1 plays important roles in the regulation of cell proliferation, adhesion, and migration, which are important factors in tumor formation and metastasis. However, these cellular behaviors are also important in many physiological processes, for instance, wound healing. Yang et al. [86] investigated the regulatory role of circAMOTL1 in wound repair and showed that injection of circAMOTL1 into wounds in C57BL/6xCBA mice generated by dermal punch biopsy resulted in accelerated healing. Using an in vitro system, Yang and co-workers [86] showed that stable overexpression of circAMOTL1 in two fibroblast cell lines, NIH3T3 and HGF, led to increased cell survival, migration, adhesion, and proliferation. By performing immunoprecipitation, the authors found that circAMOTL1 can be precipitated by antibodies recognizing several mitosis-associated proteins including E2F1, E2F4, EGF, AP1, ETS-1, NF1, and STAT3. The highest level of circAMOTL1 was pulled down by an anti-STAT3 antibody, suggesting the strongest interaction between these two molecules. STAT3 serves as an important signal transducer and activator of transcription. The ectopically expressed circAMOTL1 was found predominantly in the nucleus, similarly to the upregulated STAT3. It was found that the circAMOTL1 interaction with STAT3 facilitated the nuclear translocation of STAT3, where it bound to the regulatory elements of methyltransferase 3 alpha (DNMT3A) on genomic DNA. STAT3-mediated enhanced DNMT3A expression, in turn, led to methylation and silencing of the miR-17 promoter, which regulated miR-17-5p expression. Decreased miR-17-5p levels led to increased expression of fibronectin, DNMT3A, and STAT3, inducing higher rates of cell proliferation, migration, and wound repair (Figure 7). In this biological system, circAMOTL1 serves as a scaffold molecule for nuclear transport of transcription regulatory machinery, instead of just sponging miRNAs in the cytoplasm.

### 4.6. CircAMOTL1 Activates AKT Promoting Cardiomyocytes Proliferation and Survival

**Figure 8:**
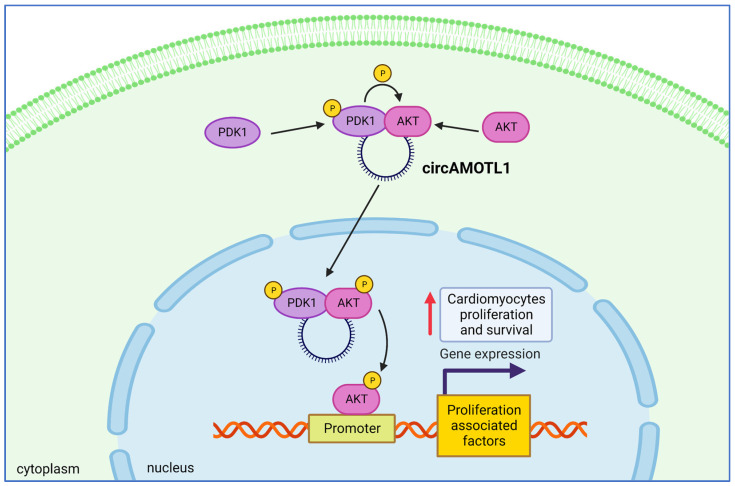
CircAMOTL1 is more highly expressed in neonatal hearts compared to mature hearts. In the cytoplasm of neonatal cardiomyocytes, circAMOTL1 serves as a docking platform for PDK1 and AKT. The formation of this ternary complex allows the phosphorylation of AKT by PDK1 and their nuclear translocation. In the nucleus, activated AKT positively regulates genes responsible for cardiomyocyte proliferation and regenerative abilities.

Myocardial injury leads to cardiomyocyte death as a consequence of oxygen and nutrient loss, impaired cardiac remodeling, and compromised regenerative abilities [104]. Several studies have described the altered expression of different circRNAs in the damaged myocardium, including circFndc3b, circ-Ttc3, and many others [105,106], which showed protective properties through accelerating the regeneration of cardiomyocytes [104,107]. CircAMOTL1 (hsa_circ_0004214) was found to be a circRNA with higher expression in neonatal hearts as compared to the mature myocardium, suggesting its potential function in cardiomyocyte survival and proliferation [87]. The overexpression of circAMOTL1 in numerous cell lines, including primary cardiomyocytes, mouse cardiac fibroblasts (MCF), endothelial cells (YPEN), and human epithelial cells (MCF-7), has been found to increase proliferation and survival, associated with decreased apoptosis. In vivo delivery of a circAMOTL1-expressing plasmid to mice with doxorubicin-induced cardiomyopathy had a cardioprotective effect. One of the most well-known and described proteins that protect cardiomyocytes from necrosis and apoptosis is AKT [108,109,110]. Typically, AKT in its inactive state is mainly in the cytoplasm, whereas the active form of AKT needs to re-localize in the nucleus to regulate the expression of proliferation and survival genes [111]. Significantly higher circAMOTL1 levels were detected in the nuclear than in the cytosolic fraction of MCF-7 cells. Similarly, in MCF-7 cells, there was an increase in the nuclear localization of phosphorylated AKT. Since phosphoinositide-dependent kinase-1 (PDK1) is a kinase that activates AKT and circAMOTL1 increases P-AKT levels in primary cardiomyocytes, Zeng et al. [87] investigated if circAMOTL1 could regulate AKT phosphorylation through interactions with PDK1. A bioinformatic simulation suggested that one circAMOTL1 molecule can serve as a scaffold for the recruitment of PDK1 and AKT simultaneously. In agreement with the bioinformatic prediction, the authors of the study elegantly showed that circAMOTL1 binds directly to PDK1 and AKT, forming a ternary complex, which facilitated AKT phosphorylation and translocation of all three molecules to the nucleus (Figure 8) [87]. This interaction resulted in increased amounts of both proteins in the nucleus, increased amounts of phosphorylated AKT, and increased levels of phosphorylated PDK1, specifically in the nucleus. The authors demonstrated that the effect of circ-AMOTL1 on cardiomyocyte protection and proliferation was dependent on AKT since the inhibition of circAMOTL1 interactions with AKT or PDK1 in MCF-7 and YPEN cells decreased the phosphorylation of AKT without affecting PDK1. Moreover, blocked activation of AKT by the AKT inhibitor triciribine led to increased apoptosis in circAMOTL1-transfected MCF-7 and YPEN cells, abolishing the effect of circAMOTL1 on cell survival. These results show that circAMOTL1 can serve as a platform for AKT activation and its nuclear translocation leading to the expression of AKT-dependent genes.

## 5. Conclusions and Perspective

Although our knowledge about the functions of AMOTL1 circRNAs is expanding, many aspects of circAMOTL1 regulation need further characterization. The mechanisms regulating the expression of hsa_circ_0004214 and hsa_circ_0000350 leading to their up or downregulation in a cancer-specific manner are unknown. However, it can be expected that they are controlled in a spatiotemporal manner through m6A modifications and RBPs. Moreover, knowing that one of the isoforms of AMOTL1 protein is depleted of the exons 2 and 3, leading to the production of a truncated protein potentially degraded [44], it is tempting to hypothesize that hsa_circ_0000350 is generated by a lariat-driven circRNA formation leading to the production of both circAMOTL1 and mRNA. A direct backsplicing mechanism with degradation of the remaining RNA likely produces other circAMOTL1 RNAs. The exact mechanism leading to the nuclear translocation of circAMOTL1 in wound healing or cardiac repair is also unknown. Interestingly, in viroids-infecting plants, nuclear import is driven by forming a C-loop on the circular RNA, which is recognized by VIRP1 that serves as a scaffold for Importin alpha-4 to mediate nuclear transport [112]. Similar mechanisms involving the formation of particular secondary structures and interactions with adaptor proteins could regulate the intracellular transport of circRNAs.

The Angiomotin gene family has emerged as regulators of many important processes. Proteins encoded by this gene family control a wide spectrum of cellular and tissue functions, including cell adhesion, migration, proliferation, mechanosensing, organ size control, angiogenesis, wound repair, heart development and regeneration, and the development of the central nervous system. The involvement of Angiomotins, including AMOTL1, in regulating the Hippo signaling pathway, gene expression, cancer development, and metastasis is of particular importance. Recently, it has become apparent that at least the AMOTL1 gene has an additional role in the production of circAMOTL1 RNAs. These non-coding RNAs have been demonstrated to serve many functions, including microRNA sponging, regulation of AMOTL1 protein expression, controlling gene expression, protein interactions, nuclear transport, and regulating post-translational proteins modification and stability (Table 1). Importantly, circAMOTL1, similarly to AMOTL1 protein, plays essential functions in physiological processes and the disease state, especially in cancer. CircAMOTL1 RNAs are implicated in the pathological mechanisms of breast cancer, cervical cancer, oral cancer, and prostate cancer, and in some cases, their expression correlates with poor prognosis. The list of disorders in which circAmotl1 is implicated will likely expand in the future, and we will soon learn more about the multiple functions of AMOTL1 gene products. It is widely recognized that alternative splicing of a given gene product can generate various protein isoforms. In parallel, we should be cautious that this process could generate circular RNAs that mediate important biological functions.

## Figures and Tables

**Table 1:** Outline of circAMOTL1 functions.

System	Model	circAMOTL1 Type	circAMOTL1 Profile	Mechanism of Action	Target	Ref.
Breast cancer	Breast cancer biopsies, SK-BR-3, MDA-MB-231, HepG2; xenografts in nude mice	hsa_circ_0004214	Upregulated in tumor samples and cell lines (oncogene)	Nuclear transport	c-Myc	[81]
Breast cancer	MDA-MB-231	hsa_circ_0004214	Upregulated in cell lines (oncogene)	Nuclear transport	AKT	[82]
Cervical cancer	HeLa, CaSki, C-33A and its xenografts in nude mice	hsa_circ_0004214	Upregulated levels in tumor samples and in cell lines (oncogene)	miRNA sponging	miR-485-5p AMOTL1	[83]
Cervical cancer	Cervical carcinoma biopsies; HeLa, SiHa, HcerEpic	hsa_circ_0004214	Upregulated in tumor samples and cell lines (oncogene)	miRNA sponging	miR-526b SIK2	[84]
Prostate cancer	DU145, PC3 and xenografts in nude mice	hsa_circ_0000350	Downregulated in tumor samples and cell lines (tumor suppressor)	miRNA sponging	miR-193a-5p PCDHA	[60]
OSCC	CAL-27, OSCC biopsies	Not known	Upregulated in tumor samples and cell line (oncogene)	miRNA sponging	miR-1294 miR-22-3p ENO1	[85]
Wound healing	NIH/3T, HGF-1, C57BL/6xCBA mice	Not known	Not tested	Nuclear transport	miR-17-5p STAT3	[86]
Cardiac repair	Primary cardiomyocytes, MCF, YPEN, MCF-7; dox-induced cardiomyopathy mouse model	hsa_circ_0004214	Upregulated in neonatal compared to mature hearts	Nuclear transport	AKT	[87]

## Data Availability

Data sharing not applicable.
